# Residential radon and characteristics of chronic obstructive pulmonary disease

**DOI:** 10.1038/s41598-022-05421-6

**Published:** 2022-01-26

**Authors:** Ana Pando-Sandoval, Alberto Ruano-Ravina, María Torres-Durán, Raquel Dacal-Quintas, Luis Valdés-Cuadrado, Jesús R. Hernández-Hernández, Angélica Consuegra-Vanegas, Cristina Candal-Pedreira, Leonor Varela-Lema, Alberto Fernández-Villar, Mónica Pérez-Ríos

**Affiliations:** 1 Department of Pneumology, Central University Teaching Hospital of Asturias, Oviedo, Spain; 2 Department of Preventive Medicine and Public Health, University of Santiago de Compostela, C/San Francisco s/n, 15782 Santiago de Compostela, Spain; 3 Consortium for Biomedical Research in Epidemiology and Public Health (CIBER en Epidemiología y Salud Pública- CIBERESP), Madrid, Spain; 4 Health Research Institute of Santiago de Compostela (Instituto de Investigación Sanitaria de Santiago de Compostela - IDIS), Santiago de Compostela, Spain; 5 Department of Pneumology, University Teaching Hospital Complex of Vigo, Vigo, Spain; 6 PneumoVigoI+I Research Group, Southern Galician Institute of Health Research (Instituto de Investigación Sanitaria Galicia Sur - IISGS), Vigo, Spain; 7 Department of Pneumology, University Teaching Hospital Complex of Ourense, Ourense, Spain; 8 Department of Pneumology, University Clinical Teaching Hospital of Santiago de Compostela, Santiago de Compostela, Spain; 9 Department of Pneumology, Avila Hospital Complex, Avila, Spain; 10 Department of Pneumology, A Coruña University Teaching Hospital, A Coruña, Spain

**Keywords:** Respiratory tract diseases, Environmental sciences, Natural hazards

## Abstract

It is not known whether residential radon exposure may be linked to the development of chronic obstructive pulmonary disease (COPD) and/or have an influence on the functional characteristics or exacerbations of COPD. The aim of this study was therefore to ascertain whether there might be an association between residential radon concentrations and certain characteristics of COPD. We analyzed COPD cases drawn from a case–control study conducted in an area of high radon exposure. Data were collected on spirometric pulmonary function variables, hospital admissions, and smoking. Radon measurements were taken using alpha-track-type CR-39 detectors individually placed in patients’ homes. All statistical analyses were performed using the IBM SPSS v22 computer software program. The study included 189 COPD cases (79.4% men; median age 64 years). The median radon concentration was 157 Bq/m^3^. No differences were found between radon concentration and sex, age or severity of breathing obstruction as measured by FEV1%. It should be noted, however, that 48.1% of patients with FEV1% < 50 had radon concentrations below 100 Bq/m^3^, as compared to 35.6% with the same severity of obstruction who had over 300 Bq/m^3^. COPD cases with radon concentrations higher than > 600 Bq/m^3^ exhibited no different characteristics in lung function. Exposure to radon does not appear to have an influence on the clinical characteristics of smokers and ex-smokers with COPD. As exposure to residential radon increases, there is no trend towards a worsening of FEV1%. Further studies are thus needed to analyze this possible association in never-smokers with COPD.

## Introduction

Diagnosis of chronic obstructive pulmonary disease (COPD) is based on tobacco use, consumption of other noxious substances, presence of respiratory symptoms, and chronic airflow limitation documented by post-bronchodilator spirometry^1^. In addition to being one of the main causes of morbidity and mortality worldwide, the disease has a substantial economic and social burden^2^. In Spain, according to the results of the EPISCAN II study, its prevalence in the population aged 40 and over is 11.8% (14.6% in men and 9.4% in women)^3^.

Although smoking habit has been associated with development of COPD since the 1950s^4^ and is its principal risk factor^5^, there are other risk factors associated with development of COPD. In fact, 25–45% of patients with COPD are never-smokers^6^, with significant variations according to the specific geographic area involved. While data from the IBERPOC study showed that 23.4% of COPD patients in Spain were never-smokers^7^, more recent data from the EPISCAN II study currently indicate that 27% of patients with COPD are never-smokers^3^. Among other risk factors, special mention should be made of genetic and environmental factors. Environmental risk factors include exposure to biomass fuels, occupational exposure to dust, powder and fumes of diverse origin, exposure to passive smoking, history of repeated lower respiratory tract infections during childhood, history of tuberculosis or chronic asthma, and premature birth or low birth weight^8,9^.

Radon is a colorless, odorless, tasteless noble gas that comes from the disintegration of uranium contained in rocks forming part of the Earth’s crust^10^. It can accumulate in closed spaces such as dwellings and workplaces^11^. It was classified as a human carcinogen in 1988 by the International Agency for Research on Cancer (IARC)^12^ and is considered to be the main risk factor for lung cancer, after smoking. Though there are different radon isotopes, the most frequent and relevant from an epidemiological point of view is Rn222 which is the one we will refer throughout the manuscript. Although there are studies that have evaluated exposure to residential radon as a possible risk factor for COPD, its influence on development of COPD is not clear. That said, however, there are studies which suggest that exposure to residential radon might increase COPD mortality^13^ as well as the risk of hospital admissions in such patients^14^. In addition, residential radon exposure has also recently been postulated as an effect modifier in the appearance of COPD in never-smokers and ex-smokers^15^. Notwithstanding these data, there is hardly any scientific evidence on the possible influence of radon on lung function or the number of hospital admissions due to COPD exacerbation.

The main aim of this study was thus to ascertain the characteristics of patients with COPD in a residential-radon-prone area, and analyze whether such exposure might be associated with pulmonary function variables, hospital admissions, and COPD exacerbations.

## Materials and methods

### Study design and setting

We conducted a multicenter, hospital-based, case–control study, fundamentally in Galicia, a designated high-risk radon exposure area^16^. Cases and controls were recruited from 5 different hospitals but in this study only cases were analyzed. The full results of the case–control study have been published recently^15^. Enrollment began in May 2018 and ended in December 2019. Cases were enrolled at the referral hospitals in each health area, and were required: firstly, to have had a previous diagnosis of COPD, confirmed in the preceding 10 years by spirometric criteria (post-bronchodilator FEV1/FVC < 70%) based on the Spanish *GesEPOC* guidelines^17^; and secondly, to have undergone at least one spirometry in the 3 years immediately prior to inclusion in the study, in which the abovementioned spirometric criteria were applied. Cases having more than 10 years elapsed since COPD diagnosis were excluded to ensure, for the most part, that only the most recent cases were included. All participants were required to be over 30 years of age and to have lived at least 15 years in the same dwelling. No participants with a history of neoplasm were included. The study protocol and consent forms were approved by the Santiago de Compostela-Lugo Research Ethics Committee (REF 2017/526). Written informed consent was obtained from all participants allowing their inclusion in the study. All methods were performed in accordance with the relevant guidelines and regulations, including STROBE guidelines for the reporting of results of observational studies.

### Data-collection

The cases were enrolled and interviewed by pulmonologists taking part in the study. A personal interview was conducted, using a questionnaire that itemized tobacco use (age of initiation and cessation, number of cigarettes per day, and environmental exposure to tobacco smoke for never-smokers), and occupational exposures. We also recorded the date of diagnosis of COPD, spirometric pulmonary function variables (at diagnosis and in last available spirometry), and number of hospital admissions in the preceding two years (both overall and by COPD exacerbation).

### Radon measurement

Radon measurements were taken using alpha-track-type CR-39 detectors (RADOSYS Inc, Hungary). At the respective hospitals, these were delivered to the participants, who then individually installed and positioned them in their homes, preferably in the main bedroom, at a height of 60–180 cm off the floor and at least 15 cm from the walls, well away from all doors, windows, electric appliances and devices. There was one detector per dwelling. The track detectors were left in place for a minimum of 3 months and then sent to the Galician Radon Laboratory (School of Medicine, Santiago de Compostela, Spain) where they were read and seasonal adjustment was made to obtain the results of radon concentration. All participants were telephoned twice, to ensure that the devices had been correctly positioned and were returned once the monitoring period had ended. The Galician Radon Laboratory (www.radon.gal) is one of three Spanish facilities officially certified by the National Accreditation Body (*Entidad de Acreditación Nacional-ENAC*) for measurement of indoor radon. The laboratory strictly follows the requirements enacted by the International Standard entitled “General requirements for the competence of testing and calibration laboratories (ISO/IEC 17025:2017)”. These standards include different forms of quality control and reproducible procedures. All participants were informed by letter about their radon concentration.

### Statistical analysis

We first carried out a descriptive analysis of COPD characteristics. We then used the Mann–Whitney test to compare radon measures and sex as a grouping variable, and the Kruskal–Wallis test to perform a comparison by age, with radon measurements being established in three categories (< 100 Bq/m^3^; 100–299 Bq/m^3^; ≥ 300 Bq/m^3^). Bivariate logistic regression was applied to assess the association between radon concentrations according to the three pre-defined categories -with the spirometric variable FEV1% grouped into two categories (FEV1% ≥ 50% and FEV1% < 50%)- and admissions due to COPD exacerbation. The Kruskal–Wallis test was used to compare radon measurements broken down into the three pre-defined categories, and functional worsening, as measured by FEV1%, between the first and last spirometry readings available. Lastly, we performed a descriptive analysis of all cases whose dwellings had radon concentrations above 600 Bq/m^3^. All analyses were performed using the IBM SPSS v22 computer software program (IBM, Armonk, NY, USA).

## Results

The study covered a total of 189 cases. The breakdown showed that: the median age was 64 years (25^th^–75^th^ percentile, 60–68 years); there was a predominance of males among the cases enrolled (79.4%); and most of the cases were ex-smokers or active smokers, and only 2 cases corresponded to never-smokers. The median residential radon concentration was 157 Bq/m^3^ (25^th^–75^th^ percentile, 93–309 Bq/m^3^). The majority of the cases included had been diagnosed with COPD at some time during the 3 years prior to their inclusion, with a median FEV1% of 55.5% (25^th^–75^th^ percentile, 40.0–70.9%), and almost half the participants reported at least one hospital admission due to COPD exacerbation in the preceding 2 years. Table 1 shows the main characteristics of the COPD cases included.

**Table 1:** Description of COPD cases included.

Variable	N (%)
**Sex**	
Men	150 (79.4)
**Age**	
Mean	63.5
Median (25^th^–75^th^percentile)	64 (60–68)
Range	47–85
Smoking status*	
Never-smokers	2 (1.1)
Ex-smokers	126 (67.0)
Current smokers	60 (31.9)
**Years living in the same dwelling**	
< 15	4 (2.2)
15–19	28 (15.1)
≥ 20	153 (82.7)
**Indoor radon exposure (Bq/m**^**3**^)*	
Mean	245
Median (25^th^–75^th^ percentile)	157 (93–309)
Range	26–1663
Years with COPD*	
3 or less	110 (59.1)
4 to 6	43 (23.1)
7 or more	33 (17.7)
**Time between last spirometry and date of inclusion**	
< 1 year	158 (85.4)
1–3 years	25 (13.5)
> 3 years	2 (1.1)
**FEV1% obtained in last spirometry**	
Mean	55.06
Median (25^th^–75^th^ percentile)	55.50 (40.07–70.87)
**Hospital admissions due to COPD exacerbation in preceding 2 years**	
0	99 (52.7)
1	44 (23.4)
2	23 (12.2)
≥ 3	22 (11.7)
**Hospital admissions due to any cause except COPD in preceding 2 years**	
0	146 (76.1)
1	26 (13.8)
2	13 (6.9)
≥ 3	6 (3.2)

*There was one participant with unknown smoking status; 7 participants had no indoor radon measurement; 3 patients had no date of diagnosis recorded.

**Figure 1:**
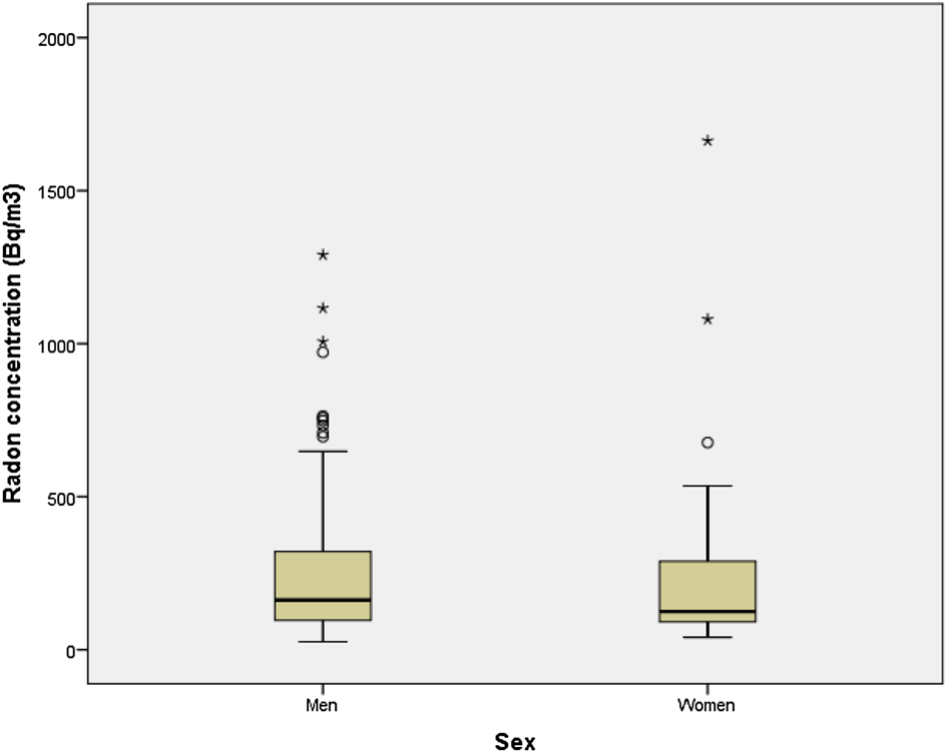
Residential radon concentrations by sex.

**Figure 2:**
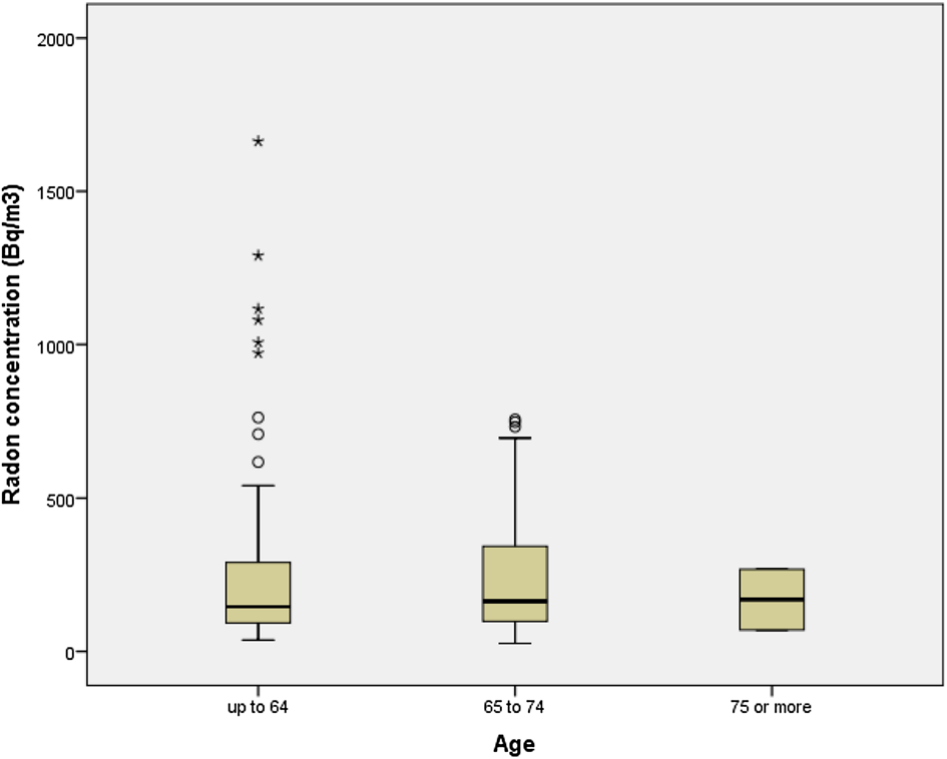
Residential radon concentrations by age.

Figure 1 shows the relationship between residential radon concentrations and sex, without a significant association being in evidence (*p* = 0.39). No differences were found in age in relation with the radon levels established in the three categories, as can be seen in Fig. 2 (*p* = 0.57). On analyzing radon concentration divided into three categories (< 100 Bq/m^3^, 100–299 Bq/m^3^ and ≥ 300 Bq/m^3^), no significant differences were observed in terms of severity of obstruction as measured by FEV1% or COPD cases who had lived more than 30 years in the same dwelling. Nonetheless, as radon concentration increased, the percentage of patients with FEV1% < 50% decreased. Similarly, as Table 2 shows, there was no evidence of a higher number of admissions due to COPD exacerbation in patients with higher residential radon concentration. Spirometric data at the diagnosis of COPD were available in 84 cases: in this group, functional worsening (as measured by FEV1% at diagnosis-FEV1% at inclusion in the study) was analyzed by reference to the residential radon concentration, with decreases of 11% being observed in the < 100 Bq/m^3^ group and 14% in the ≥ 300 Bq/m^3^ group.

**Table 2:** Relationship between radon, FEV1% in last spirometry and admissions due to COPD exacerbation in preceding 2 years.

	Radon concentration (Bq/m^3^)			*p*-value (chi-squared)
	< 100	100–299	≥ 300	
**FEV1% last spirometry (all patients)**				
≥ 50	27 (51.9)	48 (57.1)	29 (64.4)	0.460
< 50	25 (48.1)	36 (42.9)	16 (35.6)	
**FEV1% last spirometry (patients who lived more than 30 years in the same dwelling)**				
≥ 50	14 (51.9)	25 (56.8)	16 (66.7)	0.554
< 50	13 (48.1)	19 (43.2)	8 (33.3)	
**Admissions due to COPD exacerbation in preceding 2 years**				
0	28 (53.8)	47 (56.0)	21 (46.7)	0.465
1	12 (23.1)	19 (22.6)	11 (24.4)	
≥ 2	12 (23.1)	18 (21.4)	13 (28.9)	

Lastly, the characteristics of COPD cases with the highest residential radon concentrations (> 600 Bq/m^3^) were analyzed. Table 3 shows the characteristics of the 15 COPD patients with a residential radon concentration higher than 600 Bq/m^3^: their median age was 63 years, and their median FEV1% was higher than the total of the cases included, i.e., 74%. There was no evidence of an increase in hospital admissions due to COPD exacerbation in the preceding 2 years in this subgroup of COPD patients.

**Table 3:** Description of COPD cases with measurement of residential radon > 600 Bq/m^3^.

Sex	Radon concentration (Bq/m^3^)	Uncertainty of radon concentration (Bq/m^3^)	Tobacco habit	Years since COPD diagnosis	FEV1% in last spirometry	Number of COPD admissions due to exacerbation in preceding 2 years
Woman	1080	144	Smoker	1	73.30	1
Man	648	92	Smoker	0	75.20	1
Man	709	100	Smoker	0	73.00	0
Woman	1663	236	Smoker	9	65.00	2
Man	1007	142	Smoker	0	71.00	0
Man	972	138	Ex smoker	11	38.70	0
Man	1116	154	Ex smoker	3	35.20	0
Man	763	108	Ex smoker	0	69.50	0
Man	748	106	Ex smoker	0	91.00	0
Man	733	104	Ex smoker	6	83.00	0
Man	618	88	Ex smoker	7	82.00	0
Man	696	98	Ex smoker	6	74.30	0
Man	1290	184	Ex smoker	0	78.40	1
Man	757	108	Ex smoker	5	80.00	0
Woman	677	96	Ex smoker	0	77.40	1

## Discussion

This study is the first to analyze whether individual exposure to residential radon may have some influence on the clinical and functional characteristics of COPD. In general, it shows that exposure to radon does not appear to influence the clinical characteristics of smokers and ex-smokers with COPD. The relationship did not prove to be statistically significant: as exposure to indoor radon increased, there was no evidence of a trend towards a worsening of lung function. Subjects with Rn concentration above 600 Bq/m^3^ did not display different characteristics.

Previous studies have compared functional and clinical data between patients with smoking-related COPD and patients with biomass-fuel-related COPD. These studies have shown that airflow obstruction in patients with COPD secondary to exposure to biomass smoke is more characteristic of women^18^, and furthermore that the degree of obstruction in this group, as measured by FEV1%, is less severe than that linked to smoking habit^19,20^. In our study, there was a predominance of males and a medium degree of moderate air-flow obstruction, results that are more akin to those of smoking-related COPD. It should be noted that in our study only two patients were never-smokers and almost 32% were active smokers. Moreover, since only 9 persons had smoked fewer than 10 packs/year, the number of low-intensity smokers was also very small.

COPD related with exposure to biomass smoke seems to have a slower deterioration and progression than that observed in smokers. A cohort study conducted in Mexico with a 15-year follow-up analyzed FEV1 patterns in smoking-related COPD and biomass-related COPD, and reported a significantly slower rate of FEV1 decline in the latter group^19^, though these subjects did display a worse quality of life, a greater degree of hypoxemia, and no changes in mortality compared with smokers. While our study found no significant functional worsening, as measured by FEV1% data, with the increase in exposure to residential radon, there was nonetheless a certain trend towards the possibility of this effect being present.

A further important aspect to be assessed is whether there might be differences with respect to COPD exacerbations and exacerbation-related hospital admissions. Our study found no differences in the number of hospitalizations due to exacerbation of COPD related with exposure to residential radon, nor did it observe differences in those patients who had radon measures of over 600 Bq/m^3^. Once again there would seem to be a need for studies on never-smokers with COPD, so as to be able to establish whether exposure to residential radon has an effect on such patients. One study revealed a higher risk of respiratory hospitalizations, though not of mortality, among never smokers with COPD versus smokers with COPD^21^. In contrast, a Canadian cohort study found no differences in respiratory exacerbations in the preceding year between the two groups^22^. The number of exacerbations in never-smokers with COPD seems to be higher^23^, yet we have no data on differences in treatment in the two groups and are only too aware of the great importance of exacerbations in disease progression and their implication for treatment.

COPD is a chronic disease that appears after a long period of exposure of the pulmonary parenchyma to toxic particles, such as irritants deriving from tobacco smoke^1^, which cause chronic inflammation and lead to the destruction of the pulmonary parenchyma and the narrowing of the respiratory airways. The differences in type of inflammation and gene expression between smoking-related COPD and COPD in never-smokers have not been fully elucidated. Inflammation in the case of COPD in smokers is usually predominantly neutrophilic, whereas, in the case of COPD in non-smokers exposed to biomass, it is more likely to be eosinophilic^24^. One study that analyzed induced sputum in COPD patients of similar severity, found a predominance of eosinophilic inflammation in subjects exposed to biomass^25^, while another study conducted on COPD in non-smokers found lower IL-6 and IL-8 concentrations in sputum than it did in COPD patients who were smokers^26^. From a biological standpoint, it seems logical that radon decay products might be implicated in the development of COPD. The lung parenchyma is the organ that receives the highest dose of alpha radiation in a person chronically exposed to radon^27^. In COPD, inflammatory response leads to the increase in IL-6 and IL-8 that can regulate tumor growth factors^28^. Furthermore, alpha particles have been shown to induce the production of inflammatory mediators, such as IL-8 in pulmonary parenchyma^29^. This inflammatory pathway is common in COPD and lung cancer, and could be activated in both diseases by exposure to alpha particles deriving from radon, something that lends biological plausibility to this association.

Some studies have evaluated exposure to residential radon as a possible COPD-related risk factor. A systematic review published in 2020 reported a possible trend in this association but was unable to arrive at a definitive conclusion^30^. Other studies have evaluated its association with mortality or exacerbation-related hospital admissions. A cohort study undertaken in the USA^31^ reported an association between exposure to residential radon and radon mortality, and another ecological study conducted in Galicia^14^ found evidence of an association between radon concentrations and hospital admissions due to COPD. Recently, the results of this case–control study have been published, which indicate that exposure to residential radon increases the risk of COPD in smokers with an additive synergistic effect^15^.

The principal advantage of our study is that, apart from specifically measuring radon in the homes of all participants, it is the first to analyze the effect of exposure on the clinical characteristics of the disease. Furthermore, its multicentre nature lends it external validity, and the fact of its having been conducted in a high-radon-exposure area would make it easier to obtain possible associations where these existed.

Some of the limitations of this study are its use of prevalent cases rather than strictly incident cases (though 50% of cases had been diagnosed with COPD in the preceding 3 years) and the low number of cases among never-smoker COPD patients. Furthermore, we were unable to access other data which might have been interesting to analyze, such as diffusing capacity, clinical-symptomatology questionnaires, radiologic findings in high-resolution computed tomography or inhalation therapy. A further limitation is that we do not have information about radon exposure at work. Though most of our participants were retired at the time of inclusion, it cannot be disregarded that some of them might be exposed to high radon concentrations at work if we consider that this study has been performed in a radon-prone area.

In conclusion, on comparing smokers and ex-smokers with COPD, radon concentrations would not appear to differ between men and women or between different ages at diagnosis. There seems to be no relationship between exposure to radon and worse lung function as measured by FEV1%. Patients exposed to more than 600 Bq/m^3^ do not appear to present with different disease characteristics. There is a need, not only for more studies to analyze this possible association in the never-smoker population with COPD, but also for studies with a larger sample size that would enable the results of this study to be confirmed. All such studies would have to be supplied with and be based on radon measurements taken in participants’ homes.
